# Facile and Controllable Preparation of Poly(St-*co*-MMA)/FA Microspheres Used as Ultra-Lightweight Proppants

**DOI:** 10.3390/ma14237390

**Published:** 2021-12-02

**Authors:** Tao Chen, Yanan Sang, Yuxin Zhou, Liudi Ji, Xiaobing Han, Peng Hu, Pengpai Miao, Jie Gao, Yuan Zhao

**Affiliations:** 1 Hubei Key Laboratory of Radiation Chemistry and Functional Materials & Non-Power Nuclear Technology Collaborative Innovation Center, Hubei University of Science and Technology, Xianning 437100, China; taochen518@163.com (T.C.); sangyanan2021@163.com (Y.S.); zhouyx21@lzu.edu.cn (Y.Z.); jiliudi@126.com (L.J.); hanxiaobing@hbust.edu.cn (X.H.); penghoo@hust.edu.cn (P.H.); zzu666666@126.com (P.M.); 2 School of Pharmacy, Hubei University of Science and Technology, Xianning 437100, China

**Keywords:** ultra-lightweight proppants, styrene, methyl methacrylate, fly ash, suspension polymerization

## Abstract

Hydraulic fracturing is an important technology for the exploitation of unconventional oil or gas reservoirs. In order to increase the production of oil or gas, ultra-lightweight proppants with a high compressive strength are highly desirable in hydraulic fracture systems. In this work, a new type of ultra-lightweight proppant, poly(styrene-*co*-methyl methacrylate)/fly ash (poly(St-*co*-MMA)/FA) composites with a high compressive strength were prepared via in situ suspension polymerization. The Fourier transform infrared (IR) and X-ray powder diffraction (XRD) analyses confirmed that the poly(St-*co*-MMA)/FA composites were successfully prepared. The morphology analysis indicated that the composite microspheres show good sphericity, and FA powder was evenly dispersed in the matrix. The apparent density of the microspheres was between 1 and 1.3 g/cm^3^, which is suitable for hydraulic fracturing. Furthermore, the compressive strength and thermostability were dramatically improved with the incorporation of FA, which could withstand high pressures and temperatures underground. The obtained poly(St-*co*-MMA)/FA composite microspheres are promising for application as an ultra-lightweight (ULW) proppant in oil or gas exploitation, which provides a new approach for the design of high performance proppants.

## 1. Introduction

Hydraulic fracturing is widely used to increase the efficiency of oil and gas exploitation, especially in low permeability oil and gas wells [1]. Proppants are solid, spherical, millimeter-sized particles which play a critical role in the hydraulic fracturing of oil and gas, especially in the development of non-conventional oil and gas resources [2,3]. The quality of the proppant and the pattern of their placement directly determines the stimulation efficiency of the hydraulic fracturing treatment [4,5]. Proppants should have sufficient strength to keep cracks open that will form channels to make the oil and gas flow through conveniently, causing the well production to be increased significantly [6]. However, traditional proppants such as quartz sand [7], walnut shells, gravel- and resin-coated sands [8,9], and sintered ceramic [10,11,12,13] are prone to settling in hydraulic fracturing operations because of their high density, which seriously affects the efficiency of oil and gas exploitation. Therefore, it is significant to develop ultra-lightweight (ULW) proppants with good performance [3,14]. ULW proppants are 25–60% lighter than traditional proppants, but strong enough to withstand fracture closure stresses [15]. ULW proppants have a lower settling velocity and longer transportation distance than the same size of ceramic and resin-coated sand proppants, which results in a higher fracture conductivity [16,17].

The density of proppants depends on the composition, which can be adjusted by choosing the most suitable materials [18]. In our previous study, a series of organic–inorganic composite microspheres were prepared by suspension polymerization and used as ULW proppants, such as PMMA/AG [19], PMMA/SF [20,21], PMMA/FA [22], and PS/graphite [23]. However, only the PMMA or PS were used as the organic component in these proppants. As we all know, PMMA is an important material with a high modulus and outstanding chemical resistance, but it has a relatively low abrasion resistance. On the contrary, PS has a relatively low modulus but exhibits greater tensile strength and a high abrasion resistance. The co-polymerization of St and MMA can compensate for the defects of a single polymer material, which possess excellent properties and can be widely used in many fields [24].

In order to improve the mechanical strength of the polymers, inorganic fillers would be a good choice, as they can reinforce the mechanical strength dramatically. Fly ash (FA) is an important inorganic filler which is widely used in industrial production. Thus, the comprehensive utilization of the waste fly ash is urgent and significant to the long-term sustainable development of industrial production and environmental protection [25]. That is, the effective utilization of fly ash not only solves the problem of environmental pollution, but also produces high value-added products. It can improve the physical and mechanical performance of the composites with its incorporation in the polymer matrix [26].

In this work, poly(St-*co*-MMA)/FA composite microspheres with an ultra-low density and outstanding mechanical and good thermal stability were prepared via in situ suspension polymerization, exhibiting promising applications as ULW proppants. This kind of proppant would not only enhance the efficiency of oil and gas production, but can also realize the value-added utilization of fly ash, thus turning waste into treasure and harm into profit.

## 2. Experimental

### 2.1. Materials

Fly ash (FA, about 5000 mesh) was provided by HSBC New Materials Co., Ltd., Zhengzhou, China. Styrene monomers (98 wt.%, St), divinyl benzene (DVB), and stearic acid (SA) were purchased from Shanghai Macklin Biochemical Technology Co., Ltd. (Shanghai, China). Methyl methacrylate (MMA), benzoyl peroxide (BPO), γ-aminopropyltriethoxysilane (APTES), and methanol were purchased from Sinopharm Chemical Reagent Co., Ltd. (Shanghai, China). MgCl_2_·6H_2_O, NaOH, and polyvinyl alcohol (PVA) were purchased from Tianjin Fuchen Chemical Regents Factory (Tianjin, China). Deionized (DI) water was used throughout the experiments.

### 2.2. Modification of FA

A total of 120 mL toluene was added into a 250 mL flask as dispersion medium, then a certain amount of FA and APTES were added and reacted at 90 °C by mechanical stirring for 2 h. The selected weight ratio of APTES to FA was 4:100. Then, the SA was added and dispersed in the mixture and continued to reflux at 90 °C by mechanical stirring for 2 h. The selected weight ratio of SA to FA was 4:100. The product was filtered under vacuum, washed successively with deionized water and absolute ethanol several times, then dried at 60 °C for 12 h.

### 2.3. Synthesis of poly(St-co-MMA)/FA Composite Microspheres via In Situ Suspension Polymerization

**Figure 1:**
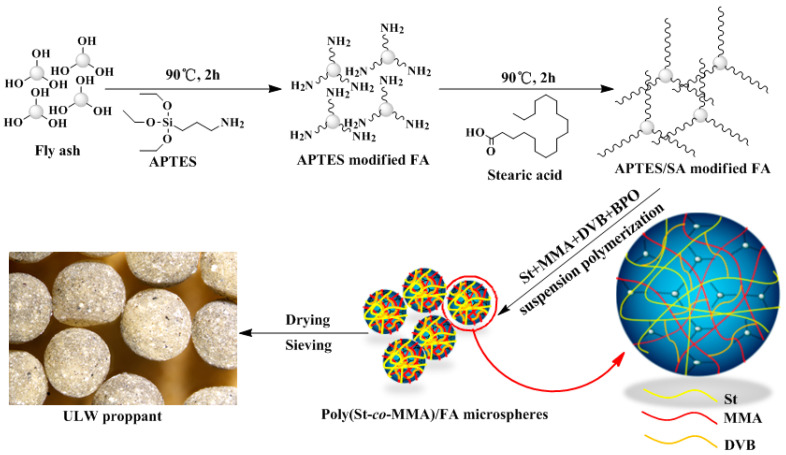
Schemes of the synthetic process of poly(St-co-MMA)/FA composite microspheres.

A typical preparation procedure is described as follows (Figure 1): 0.4 g of PVA was added to deionized water (80 mL) and stirred at 60 °C for 0.5 h under atmosphere. Subsequently, 4.0 g of MgCl_2_·6H_2_O and 1.6 g of NaOH were introduced into the mixture with a vigorous stirring for 10 min. Meanwhile, 0.3 g of BPO, 3.0 g of DVB, and a certain amount of modified FA were dissolved and dispersed in 10.0 g of MMA and 10.0 g of St in a beaker. Next, the mixture was added to the flask, The stirring speed was controlled at 290 r/min and the mixture was heated to 78 °C for 1 h, then maintained at 85 °C for 2 h and 90 °C for 2 h. The product was obtained by filtration, then washed with 60 mL of deionized water and 60 mL of methanol. Finally, the obtained poly(St-*co*-MMA)/FA composite microspheres were dried for 12 h at 60 °C. As a contrast, the pure poly(St-*co*-MMA) microspheres were prepared through a similar procedure without adding modified FA. The composites microspheres with a particle size of 20–40 mesh were selected and used as the research object.

### 2.4. Characterizations

The FTIR spectrum of composites microspheres was obtained with a NICOLET 5700 spectrometer (Thermo Fisher Nicolet, Waltham, MA, America) in the range 400–4000 cm^−1^ using the KBr pellet technique. The microstructure and surface morphology of these particles were obtained by a Binocular Microscope (Olympus Cx43). The thermogravimetry analysis (TGA) was conducted via a NETZSCH TG 209F3 instrument (NETZSCH Scientific Instruments Trading (Shanghai)Ltd., Shanghai, China) under N_2_ atmosphere with a heating rate of 10 °C·min^−1^ between 30 and 600 °C. Bulk density and apparent density were calculated by the ratio of mass to volume with a 50 mL pycnometer based on the standard of SY/T5108-2014. The compressive performance was tested with an electro-hydraulic servo universal testing machine (Jinan Hengsi Instruments Co., Ltd., Jinan, China). The breakage ratio was measured by referring to the SY/T5108-2014 standards. For the compressive strength test, 2 g samples were used and the average of three tests was taken. After crushing, the breakage ratio of the particles was calculated by the following formula:

*f=* (*Wc/W*_0_) ∗ 100%

where *Wc* is the weight of the crushed sample (g) while *W*_0_ is the weight of the sample (g) before crushing.

## 3. Results and Discussion

### 3.1. FTIR Analysis

**Figure 2:**
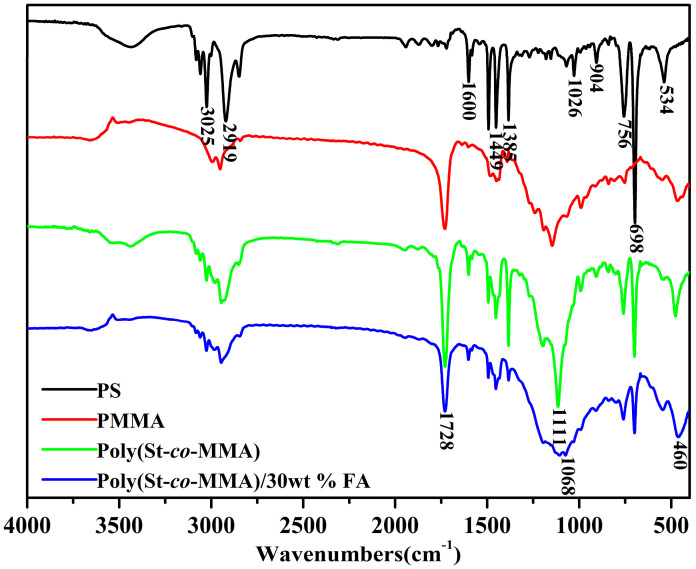
FTIR spectra of PS, PMMA, poly(St-*co*-MMA), and poly(St-*co*-MMA)/30 wt.% FA microspheres.

Figure 2 shows the FTIR spectra of the PS, PMMA, poly(St-*co*-MMA), and poly(St-*co*-MMA)/30 wt.% FA composites. The signal at 1449 cm^−1^ belongs to the bending vibration of -CH_2_- deriving from St [23]. The peak at 1728 cm^−1^ was ascribed to C=O stretching vibration, which revealed the existence of C=O groups in MMA [19]. The peak at 1068 cm^−1^ was attributed to Si–O stretching vibration. For the poly(St-*co*-MMA) copolymer, peaks at 3025 cm^−1^ and 2919 cm^−1^ indicate the C–H stretching vibration in St and MMA, respectively. A strong peak at 1728 cm^−1^ represents the C=O stretching vibration; the C–O stretching vibration in the range of 1000–1200 cm^−1^ can be assigned to the MMA. Peaks at 700–760 cm^−1^, corresponding to the C–H bending vibration, belong to the mono-substituted benzene ring, and the peak at 1500 cm^−1^ belongs to the C=C stretching of the benzene ring in St. For the poly(St-*co*-MMA)/30 wt.% FA composite, except for the vibration signals of MMA and St, more signal peaks can be observed. The peaks at 3538 cm^−1^, 1035 cm^−1^, and 400–600 cm^−1^ were attributed to the -OH bending of the silicate layers, Si–O stretching, and Si–O–Si bending vibration, respectively [24,27]. These indicated the presence of FA in the final poly(St-*co*-MMA)/30 wt.% FA composite. Overall, the FTIR investigations have clearly shown that the FA was successfully incorporated in the polymer matrix [28].

### 3.2. XRD Analysis

**Figure 3:**
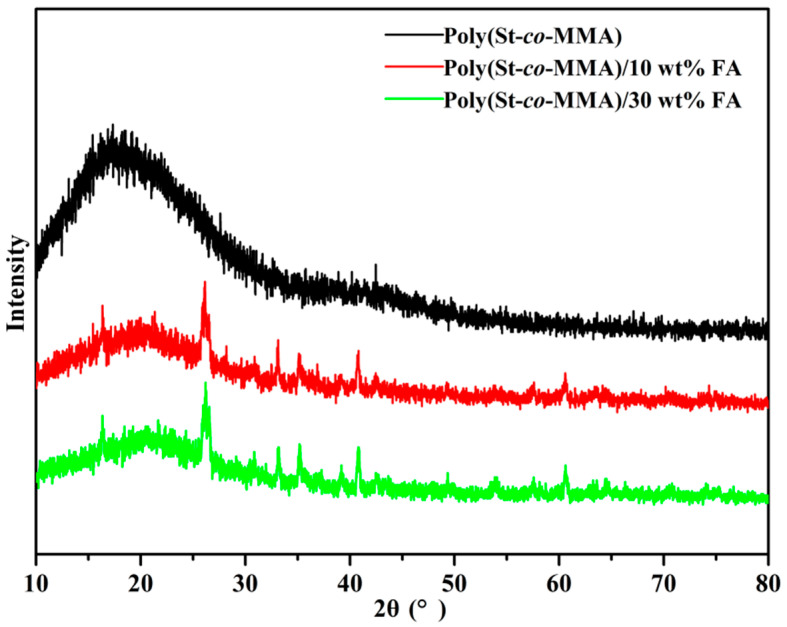
The XRD patterns of poly(St-*co*-MMA), poly(St-*co*-MMA)/10 wt.% FA, and poly(St-*co*-MMA)/30 wt.% FA.

X-ray diffraction (XRD) was used to determine the crystalline phase of the composite microspheres. Figure 3 shows the XRD patterns of poly(St-*co*-MMA) and poly(St-*co*-MMA)/FA microspheres. In all the samples based on the poly(St-co-MMA), a broad peak appeared between 13°–25°, which reveals that the copolymer materials are mainly amorphous. FA powder is also mainly constituted of an amorphous phase, containing only a small crystalline phase. The characteristic diffraction peak of fly ash appears at 2θ = 26.27° [29]. A weakened amorphous peak and small crystalline peaks were observed in the diffractograms of both of the poly(St-*co*-MMA)/FA microspheres. At the same time, with the increase of fly ash content, the characteristic peak strength increased, which indicated that the FA maintained its property in the polymer matrix, and the amorphous structure of the poly(St-*co*-MMA) did not change [22].

### 3.3. Morphology Analysis

**Figure 4:**
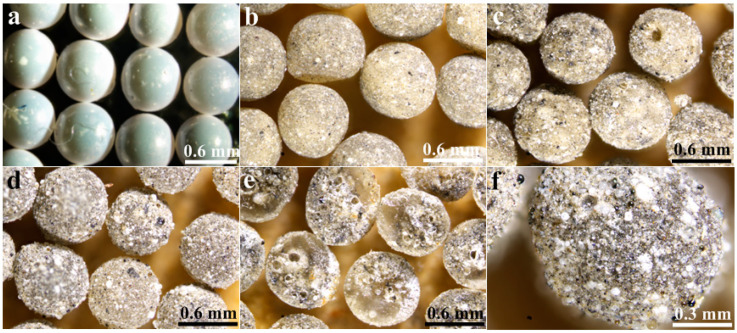
Microscope images of (**a**) pure poly(St-*co*-MMA), (**b**) 10 wt.% FA, (**c**) 30 wt.% FA, and (**d**) 50 wt.% FA microspheres. (**e**) Cross-section and (**f**) enlargement of 30 wt.% FA microspheres.

The microscope morphology of the prepared poly(St-*co*-MMA) and poly(St-*co*-MMA)/FA microspheres is shown in Figure 4. It can clearly be seen that the prepared poly(St-co-MMA) (Figure 4a) particles are a spherical shape (sphericity > 0.9) and show a uniform particle size distribution. With the increase of FA content (Figure 4b–d), the surface of the poly(St-*co*-MMA)/FA microspheres gradually roughened, and their interior (Figure 4e) and surface (Figure 4f) color gradually deepened to gray. This indicates that the fly ash was well dispersed in the polymer matrix, which will enhance the thermal and mechanical properties of the obtained composites [21,22].

### 3.4. Thermogravimetric Analysis

**Figure 5:**
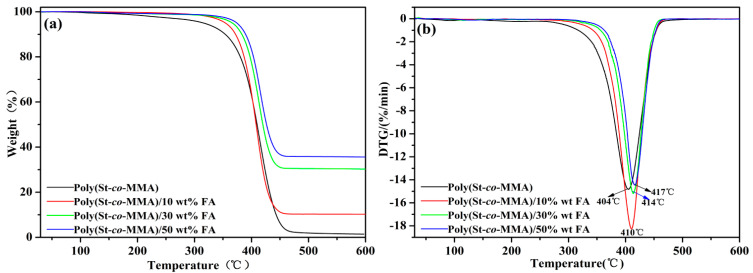
The (**a**) TG and (**b**) DTG curves of (black line) pure poly(St-*co*-MMA), (red line) 10 wt.% FA, (green line) 30 wt.% FA, and (blue line) 50 wt.% FA microspheres.

Figure 5a shows TGA plots of the pure poly(St-*co*-MMA) copolymer and poly(St-*co*-MMA)/FA composites with different FA loadings. As shown in the figure, the onset decomposition temperatures of the poly(St-*co*-MMA)/FA composites were increased in comparison with that of pure copolymer, which can be ascribed to the existence of the FA in the composite. The pure poly(St-*co*-MMA) samples had a maximum weight loss temperature of 404 °C, while the maximum weight loss temperature of the 10 wt.%, 30 wt.%, and 50 wt.% FA composites increased to 410 °C, 414 °C, and 417 °C, respectively (Figure 5b). The increase of the maximum weight loss temperature may be attributed to the heat barrier of FA platelets in poly(St-*co*-MMA). With the increase of FA content, the final weight loss of poly(St-co-MMA)/FA microspheres was lower than that of pure poly(St-*co*-MMA) microspheres; this results illustrates that FA improved the thermal stability of the poly(St-*co*-MMA) matrix [30]. This may be viewed as the good distribution of FA powder and the tortuous path in the hybrid, which hinders the diffusion of the volatile decomposition compared to that in pure poly(St-*co*-MMA). Thus, the incorporation of FA particles within the poly(St-*co*-MMA) copolymer matrix could significantly enhance the thermal stability [24,31].

### 3.5. Density Analysis

**Figure 6:**
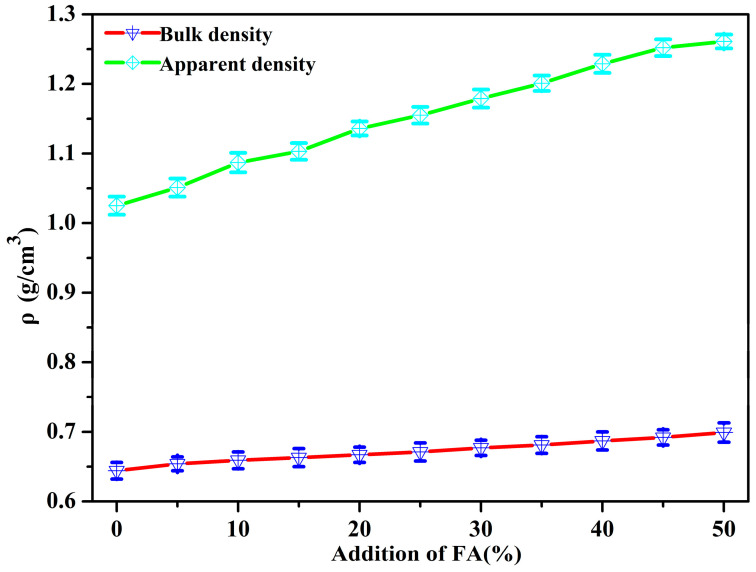
Apparent density and bulk density of poly(St-*co*-MMA)/FA microspheres.

Bulk density and apparent density are important parameters to evaluate the performance of proppants. Figure 6 displays the variation of apparent density and bulk density of poly(St-*co*-MMA)/FA microspheres with different contents of FA. Both the apparent density and bulk density revealed an increasing trend with the increase of FA content. All of the composite microspheres had an ultra-low density with a bulk density less than 0.7 g/cm^3^, and the apparent density was between 1 and 1.3 g/cm^3^, which is close to the density of water. The ultra-low density of the obtained composite microspheres was mainly ascribed to the low density of the polymer matrix (the density for St and MMA is 0.90 and 0.94 g/cm^3^, respectively) and the low density of micrometer-sized FA (5000 mesh). Thus, the composite particles are lightweight and can achieve a partial monolayer distribution in hydraulic fracturing, which could greatly reduce the proppant concentration per unit area of a fractured well, leading to high fracture conductibility with a low concentration range [32]. These results indicate that the poly(St-*co*-MMA)/FA microspheres have a potential application as ULW proppants.

### 3.6. Crushing Rate

**Figure 7:**
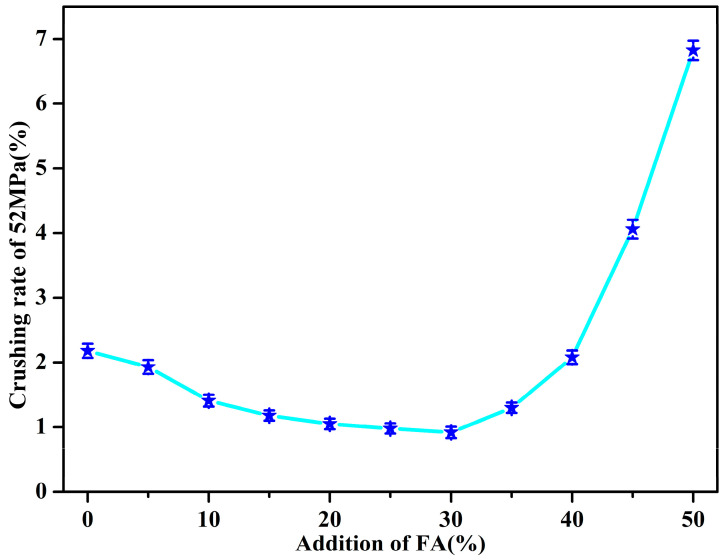
Crushing rate at 52 MPa of poly(St-*co*-MMA)/FA microspheres.

To adapt to the working environment, a proppant also needs to have excellent compressive strength; thus the crushing rate under 52 MPa of pressure was used as the evaluation standard. As shown in Figure 7, the crushing rate decreased at first and then increased with the increasing FA content. The crushing rate decreased to 0.92% when the content of FA was 30 wt.%. However, the crushing rate increased to about 6.98% when the content of FA was up to 50 wt.%, which reveals that the poly(St-*co*-MMA)/FA microspheres have a good crush resistance with 30 wt.% of FA. The reason for this phenomenon is the high interfacial shear strength between the poly(St-*co*-MMA) matrix and FA that forms from the bonding of cross-links which cover or screen the FA. Consequently, this prevents the expansion of the cracks inside the material, while the expansion of a crack can be transferred due to the good bonding between the poly(St-*co*-MMA) matrix and FA. Furthermore, the incorporation of the hard FA powders into the poly(St-*co*-MMA) matrix improves the stiffness of the composites by restricting the movement of the matrix molecular chain [33]. However, the polymer is not enough to encapsulate FA when the FA content exceeds 30 wt.%, which will weaken the intermolecular stress transfer of the polymer, thus reducing the compressive strength.

### 3.7. Comparison of Different Types of Proppants

The results of the sphericity, apparent density, bulk density, and crush resistance tests of various types of proppants are presented in Table 1. The sphericity of poly(St-*co*-MMA)/FA was close to 1, which is better than the proppants based on walnut shells (0.65) [34], quartz sand (0.68) [4], and ceramic (0.8) [35]. Higher sphericity leads to higher proppant-pack porosity, which could increase the fracture conductivity of proppants. According to the literature, there are several ways to decrease the proppant density. The first method is to select a material with a lower density, such as walnut shells and organic polymeric materials [34]. Even though such materials would penetrate deeper into the fracture, their low sphericity or strength limits their applicability to relatively low closure pressures. The second method to make lightweight proppants is to introduce a microporous structure into the proppant, such as that in resin-coated porous ceramic [36] and inorganic polymer proppants [37]. These advanced ceramic proppants have a higher strength/weight ratio than conventional ceramic proppants. However, too many holes will reduce the strength of the proppant. The third method of reducing the density of a proppant is coating [4,34,38,39]. However, due to the small content of resin on the proppant surface, it has little contribution to reducing the density of the proppant. The last way to reduce the density of a proppant is by doping inorganic minerals into organic polymers. Suspension polymerization is an effective method for preparing organic–inorganic composite microspheres. Several typical ULW proppants, such as PMMA/AG [19], PMMA/FA [21], and PS/graphite [23] composite microspheres, were prepared by suspension polymerization and used as ULW proppants in our previous study. As with the poly(St-*co*-MMA)/FA ultra-lightweight proppant of this work, all of the aforementioned proppants had a high sphericity (>0.9), ultra-low density, and good compressive strength. The studied proppant has a performance comparable to that of traditional proppants, which allows the proppant to reach the width of the fracture, travel deeper, and improve the conductivity.

## 4. Conclusions

In summary, poly(St-*co*-MMA)/FA composite microspheres were successfully synthesized via an in situ suspension polymerization. The properties of the poly(St-*co*-MMA)/FA were characterized by various techniques and the results demonstrated that FA was successfully introduced into the microspheres of poly(St-*co*-MMA). All of the obtained composite microspheres had an apparent density in the range of 1–1.3 g/cm^3^, with a good sphericity >0.9, which is very suitable for hydraulic fracturing. With the incorporation of FA (FA ratio of 30%), the crushing rate decreased to 0.92% at 52 MPa and the maximum weight loss temperature increased from 404 to 414 °C. The studied proppant thus has a comparable performance to that of conventional proppants. Moreover, it also allows improvements in conductivity that are not achieved by conventional proppants because it has an ultra-low density. The preparation approach is facile and controllable, which is suitable for large-scale production. The as-prepared cross-linked poly(St-*co*-MMA)/FA composite microsphere are a promising material for application as ULW proppants in the shale oil or gas fracturing industry.

## Figures and Tables

**Table 1:** Comparison of different types of proppants.

Proppant	Sphericity	Apparent Density (g/cm^3^)	Bulk Density (g/cm^3^)	Crushing Rate (%)	Reference
Poly(St-*co*-MMA)/FA	>0.9	1.26	0.68	0.95 (52 MPa)	This work
Polymeric materials	>0.9	1.08	0.66	2.00 (52 MPa)	34
Walnut shells	0.65	1.25	0.77	1.50 (52 MPa)	34
Resin-coated porous ceramic	0.8	1.75	1.19	7.00 (52 MPa)	34
Quartz sand	0.68	2.65	1.60	17.50 (52 MPa)	4
Resin-coated sand	0.70	2.55	1.56	5.50 (52 MPa)	4
Ceramic	0.8	3.27	1.84	0.20 (52 MPa)	35
Porous proppant	0.8	1.25		6.95 (52 MPa)	36
PS/MWCNT	0.9	1.05	0.695		1
Inorganic polymer proppant	1	1.91	1.28	21 (52 MPa)	37
Epoxy resin coated ceramic	0.9	2.27	1.32	1.16 (69 MPa)	38
Second grade bauxite ceramic	1	3.43	1.89	3.6 (52 MPa)	5
Resin-wrapped proppant	0.9	1.29	0.86	9.0 (52 MPa)	39
PMMA/AG	>0.9	1.099		0.5 (69 MPa)	19
PMMA/FA	>0.9	1.135		3 (69 MPa)	22
PS/AG proppant	0.9	1.06	0.614	1.2 (53 MPa)	23

## Data Availability

Not applicable.
